# Identification of long noncoding RNAs dysregulated in the midbrain of human cocaine abusers

**DOI:** 10.1111/jnc.13255

**Published:** 2015-09-01

**Authors:** Michael J. Bannon, Candace L. Savonen, Hui Jia, Fabien Dachet, Steven D. Halter, Carl J. Schmidt, Leonard Lipovich, Gregory Kapatos

**Affiliations:** ^1^ Department of PharmacologyWayne State University School of MedicineDetroitMichiganUSA; ^2^ Center for Molecular Medicine and GeneticsWayne State University School of MedicineDetroitMichiganUSA; ^3^ Department of NeurologyWayne State University School of MedicineDetroitMichiganUSA; ^4^ Department of PathologyUniversity of MichiganAnn ArborMichiganUSA

**Keywords:** cocaine, dopamine, drug abuse, gene expression, long noncoding RNA, postmortem

## Abstract

Maintenance of the drug‐addicted state is thought to involve changes in gene expression in different neuronal cell types and neural circuits. Midbrain dopamine (DA) neurons in particular mediate numerous responses to drugs of abuse. Long noncoding RNAs (lncRNAs) regulate CNS gene expression through a variety of mechanisms, but next to nothing is known about their role in drug abuse. The proportion of lncRNAs that are primate‐specific provides a strong rationale for their study in human drug abusers. In this study, we determined a profile of dysregulated putative lncRNAs through the analysis of postmortem human midbrain specimens from chronic cocaine abusers and well‐matched control subjects (*n* = 11 in each group) using a custom lncRNA microarray. A dataset comprising 32 well‐annotated lncRNAs with independent evidence of brain expression and robust differential expression in cocaine abusers is presented. For a subset of these lncRNAs, differential expression was validated by quantitative real‐time PCR and cellular localization determined by *in situ* hybridization histochemistry. Examples of lncRNAs exhibiting DA cell‐specific expression, different subcellular distributions, and covariance of expression with known cocaine‐regulated protein‐coding genes were identified. These findings implicate lncRNAs in the cellular responses of human DA neurons to chronic cocaine abuse.

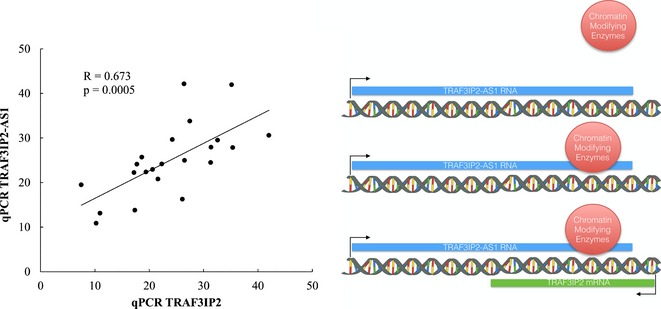

Long noncoding RNAs (lncRNAs) regulate the expression of protein‐coding genes, but little is known about their potential role in drug abuse. In this study, we identified lncRNAs differentially expressed in human cocaine abusers' midbrains. One up‐regulated antisense lncRNA, tumor necrosis factor receptor‐associated factor 3‐interacting protein 2‐antisense 1 (TRAF3IP2‐AS1), was found predominantly in the nucleus of human dopamine (DA) neurons, whereas the related TRAF3IP2 protein‐coding transcript was distributed throughout these cells. The abundances of these transcripts were significantly correlated (left) suggesting that TRAF3IP2‐AS1 may regulate *TRAF3IP2* gene expression, perhaps through local chromatin changes at this locus (right).

**Abbreviations used**
DA: dopamine
FDR: false discovery rate
GAPDH: glyceraldehyde 3‐phosphate dehydrogenase
HOTAIRM1: HOX antisense intergenic RNA myeloid 1
ISHH: *in situ* hybridization histochemistry
LINC00162: long intergenic noncoding RNA 00162
lncRNA: long noncoding RNA
MALAT1: metastasis associated lung adenocarcinoma transcript 1
MIAT: myocardial infarction associated transcript
NEAT1: nuclear enriched abundant transcript 1
NF‐kB: nuclear factor kappa‐light‐chain‐enhancer of activated B cells
PRKCQ‐AS1: protein kinase C theta‐antisense 1
PRKCQ: protein kinase C theta
qPCR: quantitative real‐time PCR
RIN: RNA integrity number
TRAF3IP2‐AS1: tumor necrosis factor receptor‐associated factor 3‐interacting protein 2‐antisense 1
TRAF3IP2: tumor necrosis factor receptor‐associated factor 3‐interacting protein 2

Drug addiction is a debilitating chronic disorder characterized by craving, compulsive use of drugs even in the face of adverse consequences, and high incidences of relapse. At a molecular level, long‐lived changes in neural gene expression arising through transcriptional and epigenetic mechanisms are thought to constitute a ‘molecular memory' that contributes to the maintenance of a drug‐addicted state (Feng and Nestler 2013). Of the different neural cell types and neural circuits implicated in the effects of drugs of abuse, perhaps none play a more central role than dopamine (DA)‐synthesizing neurons of the ventral midbrain which, though few in number, mediate many acute rewarding effects of drugs of abuse, conditioned responses to cues associated with previous drug use, and the emergence of some adverse effects upon cessation of drug use (Koob and Volkow 2010; Volkow *et al*. 2011). Recent analysis of human postmortem midbrain has revealed a molecular signature of pathophysiological changes in gene expression that are diagnostic for chronic cocaine abuse (Bannon *et al*. 2014, 2015), but our understanding of the mediators of these changes remains rudimentary.

Recent transcriptional analyses have revealed that, although only a small fraction of the human genome is translated into proteins, the majority of genomic sequence is transcribed to produce many thousands of noncoding RNAs, a large proportion of which are long noncoding RNAs (lncRNAs), RNAs > 200 nucleotides in length but lacking extended open reading frames (Lipovich *et al*. 2010; Derrien *et al*. 2012; Encode Project Consortium 2012). Emergent data suggest that lncRNAs can regulate the expression of protein‐coding genes through a striking variety of mechanisms, including locus‐specific or widespread targeting of epigenetic modifications, nucleating assembly of RNA splicing complexes, or modifying the stability or translation of specific cytoplasmic mRNAs (Guttman and Rinn 2012; Mercer and Mattick 2013; Clark and Blackshaw 2014). In the CNS, some lncRNAs show strong cell‐specificity of expression, modulate the developmental specification of individual neuronal subtypes and, most recently, have been implicated in several CNS disorders (Modarresi *et al*. 2012; Pastori and Wahlestedt 2012; Ng *et al*. 2013; Clark and Blackshaw 2014; Punzi *et al*. 2014). In contrast, we know very little about the potential role lncRNAs may play in drug abuse (Michelhaugh *et al*. 2011; Bu *et al*. 2012). As approximately one‐third of the thousands of human lncRNAs identified appear to be unique to the primate lineage (Derrien *et al*. 2012), there is a compelling rationale for studying lncRNAs in the drug‐addicted human brain as well as simpler model systems.

To address this significant gap in knowledge, this study investigated lncRNA expression in the postmortem midbrain of human cocaine abusers and well‐matched control subjects. A profile of lncRNAs dysregulated in chronic cocaine abusers was determined. LncRNAs exhibiting DA cell‐specific expression, different subcellular distributions, and covariance of expression with known cocaine‐regulated protein‐coding genes were identified. The findings are consistent with the notion that some lncRNAs may act as mediators of cellular responses to drug abuse.

## Materials and methods

### Human brain specimens

Human midbrain specimens were obtained by forensic pathologists in the course of the routine autopsy process, and de‐identified specimens were subsequently characterized as described previously (Bannon and Whitty 1997; Albertson *et al*. 2004, 2006; Michelhaugh *et al*. 2011; Okvist *et al*. 2011; Johnson *et al*. 2012; Bannon *et al*. 2014, 2015). Briefly, cause of death was determined by forensic pathologists following medico‐legal investigations evaluating the circumstances of death including medical records, police reports, autopsy results, and toxicological data. Case inclusion in the group of cocaine‐related fatalities (*n* = 11) was based on a documented history of drug abuse, a toxicology positive for cocaine and/or cocaine metabolites but negative for other drugs of abuse or CNS medications at time of death, and forensic determination of cocaine as a cause of death. Cases in the control group (*n* = 11) had no documented history of drug abuse, and tested negative for cocaine, cocaine metabolites, and other drugs of abuse or CNS medications (other than a single case with a subintoxicating ethanol level of 0.06 g/dL). Causes of death for control cases were cardiovascular accidents or gunshot wounds. Cases were not screened for the presence of nicotine or metabolites. Exclusion criteria for either group included a known history of neurological or psychiatric disorder, evidence of neuropathology (e.g., stroke, encephalitis) or chronic illness (e.g., cirrhosis, cancer), death by suicide, or an estimated postmortem interval > 20 h. To reduce variance unrelated to drug abuse, the two groups were matched (Table 1) in terms of gender, race, age, and well‐established measures of tissue sample quality (brain pH) and perimortem agonal state (RNA integrity number, RIN) (Schroeder *et al*. 2006; Stan *et al*. 2006). The use of de‐identified cadaver specimens obtained at autopsy is not defined as human subjects research and therefore exempt from regulation 45 CFR pt 46 (NIH SF424 guide Part II: Human Subjects).

**Table 1:** Characteristics of study subjects

	Control subjects					Cocaine subjects						
	Age	Race/sex	Cause of death	pH	RIN	Age	Race/sex	Cause of death	pH	RIN	Cocaine μg/mL	BE μg/mL
	66	BM	MGSW	6.7	6.9	64	BM	Cocaine intoxication	6.7	7.6	0.07	0.40
	50	BM	GSW	6.7	6.9	51	BM	Cocaine abuse	6.7	5.7	ND	0.13
	51	BF	ASCVD	6.5	6.9	52	BF	Cocaine abuse, intracerebral hemorrhage	6.3	6.6	ND	0.08
	40	BF	Dilated cardiomyopathy	6.4	6.8	34	BF	Cocaine abuse	6.4	6.2	ND	0.66
	35	BM	GSW, ASCVD	6.4	6.4	35	BM	Cocaine intoxication	6.7	7.7	0.28	5.3
	47	WM	ASCVD	6.2	6.1	46	WM	Cocaine intoxication	6.4	7.3	0.80	5.7
	45	BM	MGSW	6.3	7.5	49	BM	Cocaine abuse, aortic aneurysm	6.4	6.7	ND	0.42
	49	BM	ASCVD	6.8	7.0	59	BM	Cocaine abuse	6.6	7.3	ND	0.07
	52	BM	Hypertensive cardiomyopathy	6.3	7.0	52	BM	Cocaine abuse	6.6	5.6	0.08	0.33
	53	BM	ASCVD	6.3	7.8	52	BM	Cocaine abuse	6.5	6.5	0.29	3.0
	51	BM	Aortic dissection, hypertension	6.7	6.0	52	BM	Cocaine abuse, aortic dissection	6.3	7.2	ND	0.58
Mean (SEM)	49 (7.5)			6.5 (0.2)	6.9 (0.5)	50 (8.5)			6.5 (0.1)	6.8 (0.7)		

ASCVD, arteriosclerotic cardiovascular disease; BE, major cocaine metabolite benzoylecgonine; BF, black female; BM, black male; GSW, gunshot wound; MGSW, multiple gunshot wounds; ND, not detected; RIN, RNA integrity number; SEM, standard error of the mean; WM, white male.

### Sample processing and microarray analysis

All methodologies have been previously described in detail (Bannon and Whitty 1997; Albertson *et al*. 2004, 2006; Michelhaugh *et al*. 2011; Okvist *et al*. 2011; Johnson *et al*. 2012; Bannon *et al*. 2014, 2015). Briefly, post‐mortem samples encompassing the entire ventral midbrain (encompassing approximately plates 51–56 of DeArmond *et al*. 1989) were fresh‐frozen upon collection at autopsy, cryosectioned, and DA cell‐enriched regions finely dissected and pooled for each subject. RNA was isolated, quantified, and assessed for integrity (by RIN) using an Agilent Bioanalyzer (Santa Clara, CA, USA).

LncRNAs represented on our custom human lncRNA microarray were chosen through a process of genome‐wide computational identification and manual annotation of putative lncRNAs, as previously described (Jia *et al*. 2010). In addition, some protein‐coding genes previously shown to be affected by cocaine abuse (Bannon *et al*. 2014) were included on the microarray as positive controls. Microarray experiments were executed as previously described (Lipovich *et al*. 2012). Briefly, cRNAs were generated from each case and hybridized to a custom Agilent 4 × 44 000‐feature high‐density oligonucleotide microarray platform designed to interrogate 5586 unique putative lncRNAs (plus an additional 120 protein‐coding and housekeeping genes serving as controls), with seven 60‐mer probes assigned to each gene (Jia *et al*. 2010; Lipovich *et al*. 2012). Microarray experiments were performed with cocaine‐control matched pairs in a dye‐flip two‐color design, meaning each sample was run in quadruplicate, twice with each dye (Alexa‐647 and Alexa‐555; Invitrogen, Carlsbad, CA, USA). Microarray slides were scanned with the default Agilent protocol and the intensity of fluorescence between dyes was normalized using a Loess correction. Data across all cases and quadruplicates were quantile‐normalized and validated using MA plot density and distribution analysis (Lipovich *et al*. 2012). Approximately one‐half of all probes were detected above background in the majority of subject pairs. The entire microarray dataset is available in the Gene Expression Omnibus repository (www.ncbi.nlm.nih.gov/geo; GSE67281).

### Bioinformatics and statistics

The criteria used for selection of lncRNA transcripts for further analysis are graphically represented in Figure S1. Briefly, for this study, a putative lncRNA transcript was classified as differentially expressed in cocaine abusers versus control subjects only if the signal from all seven non‐identical microarray probes sequences was significantly changed, as determined by a two‐step mixed model anova, which utilizes both within pairs and between‐groups comparisons. A false discovery rate of 0.05 was applied to obtain final corrected *p*‐values. Of the 428 putative lncRNA transcripts meeting these criteria, 91 exhibited an average fold change in ≥ 1.3. The subset of these transcripts most strongly supported by EST data in the UCSC Genome browser (Dec 2009 hg19 assembly), as well as expression data from the Burge Brain RNA‐Seq (Wang *et al*. 2008), Sestan Brain microarray (Johnson *et al*. 2009), Allen Brain (Hawrylycz *et al*. 2012), or FANTOM5 (Andersson *et al*. 2014; FANTOM Consortium and the RIKEN PMI and CLST (DGT) *et al*. 2014) datasets served as the basis for further study. The UCSC Genome browser (Dec 2009 hg19 assembly) was also used to examine the presence of polyadenylation and pre‐RNA splicing consensus sequences in human lncRNA genes and their conservation across species.

Correlations between lncRNA abundances and cocaine metabolite levels (Table S2), or between lncRNA and protein‐coding gene transcript levels (Table 3), were calculated using Pearson's correlations. The LncRNA2 Function database (http://mlg.hit.edu.cn/lncrna2function) was used for a computational investigation of the potential functionality of lncRNAs based on patterns of co‐expression with protein‐coding genes in 19 human tissues (Table S3).

### Quantitative PCR and *in situ* hybridization histochemistry

Differential expression of six (three up‐regulated and three down‐regulated) lncRNAs from Table 2 was validated by quantitative real‐time (qPCR), as previously described (Bannon and Whitty 1997; Albertson *et al*. 2004, 2006; Michelhaugh *et al*. 2011; Okvist *et al*. 2011; Johnson *et al*. 2012; Bannon *et al*. 2014, 2015). Primers sequences are provided in Table S1. Pearson's correlations between the microarray data and qPCR data for these transcripts were determined (Michelhaugh *et al*. 2011; Bannon *et al*. 2014).

**Table 2:** Differentially regulated lncRNAs in the ventral midbrain of cocaine abusers

lncRNA	ENSEMBL gene ID	Gene location	Average fold difference	Relative abundance	Polyadenylation consensus sequence and conservation	Splice site consensus sequence and conservation	Detected brain expression	Remarks
**Up‐regulated lncRNAs**								
RP11‐309G3.3	ENSG00000272198	chr1q25.3	2.35	16.2	No	No	B, S	Intergenic. Adjacent to *IER5* (same strand)
RP1‐212P9.2	ENSG00000226852	chr1p35.3	2.03	74.1	Yes: conserved	Yes: conserved	B, F, S	Intergenic. Adjacent to *OPRD1* (same strand)
LOC101929176	ENSG00000250579	chr5p15.32	1.77	30.1	Yes: primate	Yes: mixed	S	Intergenic. Adjacent to *ADAMTS16* (same strand)
LOC100507534	ENSG00000261325	chr16q12.1	1.67	117.2	Yes: primate	Yes: conserved	B, F, S	Intergenic. Within a cluster of lncRNAs (both strands)
RP11‐109G23.3	ENSG00000260278	chr4q21.21	1.61	8.9	Yes: conserved	Yes: mixed	S	Intergenic. Adjacent to *BMP2K* (opposite strand)
HOTAIRM1	ENSG00000233429	chr7p15.2	1.53	293.5	Yes: conserved	Yes: conserved	F, S	Antisense. Regulates transcription of *HOXA1* and *HOXA2* genes
RPPH1	ENSG00000259001	chr14q11.2	1.49	22.4	No	No	B, F, S	Antisense. Component of RNAse P involved in tRNA maturation
TRAF3IP2‐AS1	ENSG00000231889	chr6q21	1.45	477.4	Yes: conserved	Yes: conserved	A, B, F, S	Antisense to *TRAF3IP2*
RP11‐552F3.9	ENSG00000267801	chr17q25.1	1.40	5.9	No	No	F, S	Antisense to *TRIM47*
RP11‐49I11.1	ENSG00000260552	chr18q12.2	1.37	4.4	Yes: primate	Yes: conserved	F, S	Antisense to *MOCOS*
RP4‐809F18.2	ENSG00000257061	chr12q24.32	1.37	8.0	No	Yes: mixed	B, S	Intergenic. Adjacent to LINC *RP4‐809F18.1* (same strand)
AC083843.1	ENSG00000259820	chr8q24.22	1.33	60.4	Yes: conserved	No	B, S	Intergenic. Adjacent to *MIR30B* and *MIR30D* (same strand)
WDR11‐AS1	ENSG00000227165	chr10q26.12	1.33	36.9	No	Yes: conserved	B, F, S	Antisense to *WDR11*
RP11‐521016.2	ENSG00000260163	chr2q14.3	1.31	56.9	Yes: primate	Yes: mixed	B, F, S	Antisense to LINC *RP11‐521016.1*
**Down‐regulated lncRNAs**								
RNF219‐AS1	ENSG00000234377	chr13q22.3	−1.31	87.8	Yes: conserved	Yes: mixed	B, F, S	Antisense to *RNF219*
RP11‐553L6.5	ENSG00000259976	chr3q13.31	−1.31	3471.8	Yes: conserved	No	B	Sense overlapping with *ZBTB20*
PRKCQ‐AS1	ENSG00000237943	chr10p14	−1.33	149.2	Yes: primate	Yes: conserved	B, F, S	Antisense to (shared 5′ region with) *PRKCQ*
RP11‐388C12.1	ENSG00000263063	chr17q25.3	−1.33	34.4	No	No	S	Intergenic. Adjacent to *FN3KRP* (opposite strand)
STX18‐AS1	ENSG00000247708	chr4p16.2	−1.35	5.1	Yes: conserved	No	B, F, S	Antisense to *STX18*
LINC00540	NA	chr13q12.11	−1.36	10.1	No	Yes: conserved	S	Intergenic. Nearest neighbors are clusters of lncRNAs
LOC100507140	ENSG00000237166	chr2q33.1	−1.37	196.1	No	Yes: conserved	B, F, S	Antisense to *AOX2P*
LINC00403	ENSG00000224243	chr13q34	−1.39	865.9	No	Yes: mixed	A, B, F, S	Sense overlapping with *SOX1*,* SNORD441*, and *RP11‐450H6.3*
RP11‐539L10.3	ENSG00000251580	chr4p16.1	−1.42	985.1	Yes: conserved	Yes: primate	B, F, S	Antisense to lncRNAs *LOC93622* and *AC093323.3*
AP001505.9	ENSG00000261706	chr21q22.3	−1.43	47.3	Yes: conserved	No	F, S	Intergenic. Adjacent to lncRNAs *LINC00163* and *LINC00162*
LOC400548	ENSG00000278214	chr16q24.1	−1.44	17.0	No	Yes: mixed	B, F, S	Intergenic. Nearest genes *FAM92B* and *CTC‐786C10.1*
LOC643763	ENSG00000274956	chr8q12.3	−1.46	1569.0	Yes: conserved	No	A, B, F	Sense intronic within single exon of *NKAIN3*
LINC01314	ENSG00000259417	chr15q25.1	−1.49	543.8	Yes: primate	Yes: conserved	A, B, F, S	Intergenic. Adjacent to *LINC00927* and *FAH* (opposite strand)
RP11‐23P13.6	ENSG00000174171	chr15q15.1	−1.50	6.1	No	No	B, F, S	Antisense to *SPTBN5*
AFAP1‐AS1	ENSG00000272620	chr4p16.1	−1.62	7.1	Yes: conserved	Yes: mixed	A, B, F, S	Antisense *AFAP1*
LINC00645	ENSG00000258548	chr14q12	−1.69	87.1	Yes: conserved	Yes: mixed	S	Antisense intronic to lncRNA *BC148262*
LINC00162	ENSG00000224930	chr21q22.3	−1.74	65.1	No	Yes: mixed	A, B, F, S	Intergenic. Adjacent to *LINC000163*, and lncRNA *AP001505.9*
LINC01010	ENSG00000236700	chr6q23.2	−1.99	36.9	Yes: conserved	Yes: mixed	B, F, S	Antisense to lncRNAs *LH15.4OC101928231* and *RP11‐557H15.3*

Relative abundance is overall fluorescence signal/background. In addition to expression data from UCSC Genome Browser, Dec 2009 hg19 chr assembly, transcript detection: A, in substantia nigra by Allen Brain Atlas microarray (Hawrylycz *et al*. 2012); B, in Burge brain RNA‐Seq (Wang *et al*. 2008); S, in Sestan brain microarray (Johnson *et al*., 2009); F in substantia nigra by FANTOM5 CAGE database (Andersson *et al*. 2014). A gene feature found in primates as well as at least one non‐primate species is denoted as conserved. Primate‐specific gene features, and mixtures of conserved and primate‐specific features, are also indicated.

Using *in situ* hybridization histochemistry (ISHH), the cellular and subcellular localization of several transcripts were examined in 14 μm sections of human midbrain using previously published methods (Bannon and Whitty 1997; Okvist *et al*. 2011). Digoxigenin‐labeled antisense or sense (control) riboprobes were transcribed (DIG RNA labeling reagents; Roche, Indianapolis, IN, USA) from cloned DNA sequences derived using the same parameters as qPCR validation experiments (Table S1). The signal was developed using anti‐digoxigenin‐alkaline phosphatase conjugated Fab fragment with nitro blue tetrazolium/5‐bromo‐4‐chloro‐3‐indolyl phosphate as substrate. Images were captured using an Olympus BX53 microscope (Center Valley, PA, USA) and 60× immersion objective and CellSens software with image deconvolution and brightness adjustment.

## Results

Cocaine‐related fatalities in this study were closely matched with drug‐free control subjects in terms of race, sex, and age (Table 1) in an effort to minimize potential variance in gene expression data unrelated to cocaine abuse. There were also no differences between groups in terms of well‐established measures of tissue sample quality (i.e., brain pH) or perimortem agonal state (i.e., RIN values) (Table 1). Post‐mortem specimens of human ventral midbrain enriched for DA neurons (Bannon *et al*. 2014) were processed in parallel through all experimental procedures and hybridized in quadruplicate to custom lncRNA microarrays as described (Lipovich *et al*. 2012) to maximize the accuracy of gene expression profiles. The dataset has been deposited in its entirety in the Gene Expression Omnibus repository (www.ncbi.nlm.nih.gov/geo; GSE67281).

LncRNA transcripts were selected for further analysis based on a series of criteria (Figure S1). Briefly, for the purposes of this study, a putative lncRNA transcript was initially classified as differentially expressed in cocaine abusers versus control subjects only if the signal intensity of all seven non‐identical microarray probes sequences was significantly different, as determined by anova with an false discovery rate of 5%. Of 5586 putative lncRNAs represented on the microarray, 428 met this criterion. To restrict subsequent analysis to the most robustly changed and well‐annotated of these transcripts, the dataset was further pared down using both a magnitude of difference threshold (≥ 1.3 average fold difference) and the requirement of independent evidence of expression in brain (see Materials and methods). Application of this stringent set of criteria yielded a final list of 32 well‐annotated lncRNAs exhibiting robust differential expression (14 were up‐regulated and 18 were down‐regulated) in the midbrains of human cocaine abusers (Table 2).

**Figure 1:**
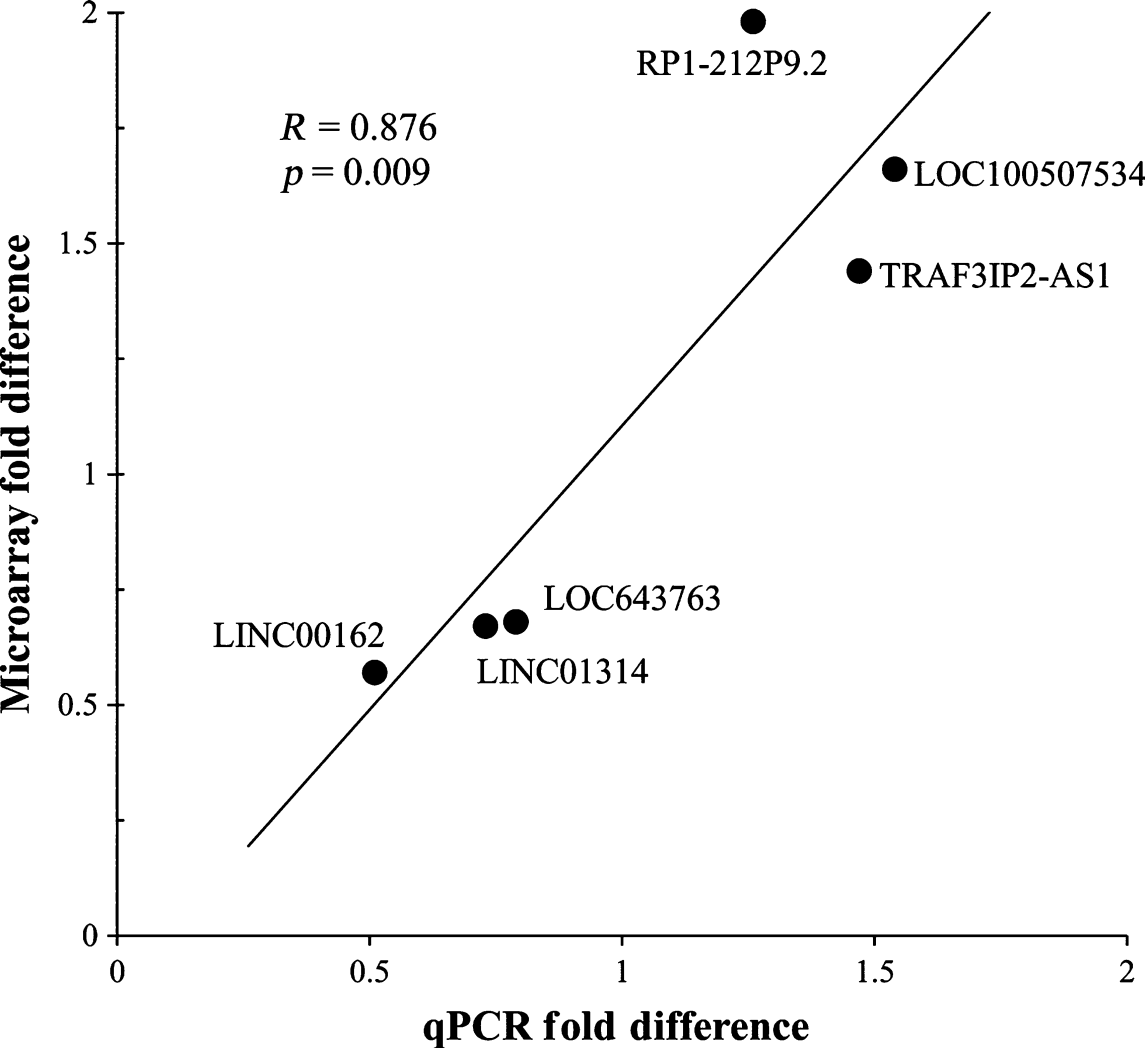
Validation of differential lncRNA expression detected by microarray. Six lncRNAs spanning a range of transcript abundances and magnitude differences were selected from Table 2 for validation by quantitative real‐time PCR. Pearson's correlation between microarray data and qPCR data is shown. Primer sequences can be found in Table S1.

Of these 32 lncRNAs, three up‐regulated and three down‐regulated transcripts, representing a range of fold‐differences and abundances, were further analyzed by qPCR. In each instance examined, qPCR data validated the custom microarray data (*p* = 0.009; Fig. 1), supporting the robust nature of the findings. Importantly, the abundances of differentially expressed lncRNAs were not correlated with subjects' levels of cocaine metabolite (Table S2), providing evidence that the recency of cocaine use was likely not a major determinant of the differential expression seen in cocaine abusers.

The features of the lncRNAs listed in Table 2 were consistent with those described in global analyses of lncRNAs (Lipovich *et al*. 2010; Derrien *et al*. 2012; Wight and Werner 2013). Approximately two‐thirds of the these differentially expressed lncRNAs included a polyadenylation consensus sequence within 100 bases of the transcript 3′ end; of these, one‐third contained primate lineage‐specific sequence, whereas two‐thirds showed sequence conservation beyond the primate lineage (Table 2). Canonical consensus sequences for pre‐RNA splicing were also found in over two‐thirds of the corresponding lncRNA genes; these were equally divided between genes with a mix of conserved and primate‐specific splice sites and genes with no primate‐specific consensus sequences (only a single gene showed primate‐specific splice site sequences exclusively) (Table 2). In terms of genomic localization, approximately one‐half of the lncRNA genes were antisense (opposite strand) to protein‐coding or other lncRNA genes; the bulk of the remaining lncRNA genes were intergenic (i.e., not overlapping with known genes) (Table 2).

**Figure 2:**
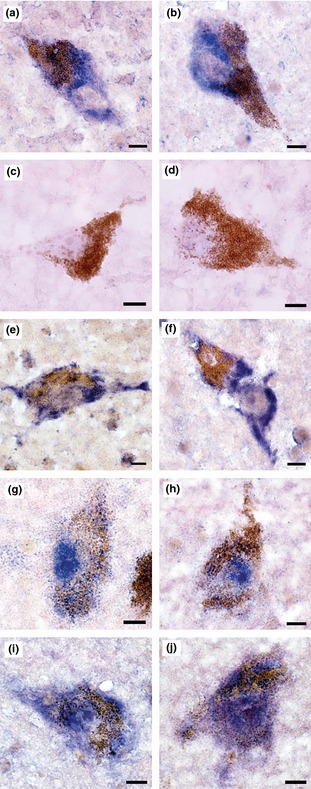
Cellular localization and subcellular distribution of selected transcripts determined by *in situ* hybridization histochemistry. **(**a, b**)** Robust expression of dopamine (DA) transporter mRNA within the processes and soma of DA neurons (also readily identifiable by their characteristic large nuclei and high intracellular neuromelanin content). (c, d) Specificity was demonstrated by the absence of signaling using a riboprobe derived from bacterial neomycin gene sequence as a negative control. (e, f) Similar to DA transporter mRNA localization, LINC00162 transcript was robustly expressed within the processes and soma of DA cells, with nuclear exclusion. (g, h) TRAF3IP2‐AS1 transcript exhibited a strong nuclear localization in DA cells. (i, j) TRAF3IP2 protein‐coding transcript distribution was distinctly different from TRAF3IP2‐AS1 transcript, and was found throughout the nucleus, cytoplasm and processes of DA neurons. Probe sequences can be found in Table S1. Images captured with a 60× objective. Scale bars equal 10 microns.

From among the six lncRNAs validated by qPCR, the cellular and subcellular localization of a down‐regulated transcript (long intergenic noncoding RNA 00162, LINC00162) and an up‐regulated transcript (tumor necrosis factor receptor‐associated factor 3‐interacting protein 2‐antisense 1, TRAF3IP2‐AS1) was examined in human ventral midbrain by means of ISHH (Fig. 2). As a positive control, the robust expression of DA transporter‐encoding transcript was visualized within the processes and soma of DA neurons (readily identifiable by their characteristic large nuclei and high intracellular neuromelanin content) (Fig. 2a and b). Specificity of the ISHH procedure was further demonstrated by the absence of signaling using a negative control riboprobe (directed against bacterial neomycin gene sequence; Fig. 2c and d). Qualitative analysis indicated that both LINC00162 and TRAF3IP2‐AS1 lncRNA transcripts were visualized nearly exclusively in DA neurons (Fig. 2e–h). Similar to DA transporter transcript, LINC00162 transcript was robustly expressed within the processes and soma of DA cells, with nuclear exclusion (Fig. 2e and f).

**Figure 3:**
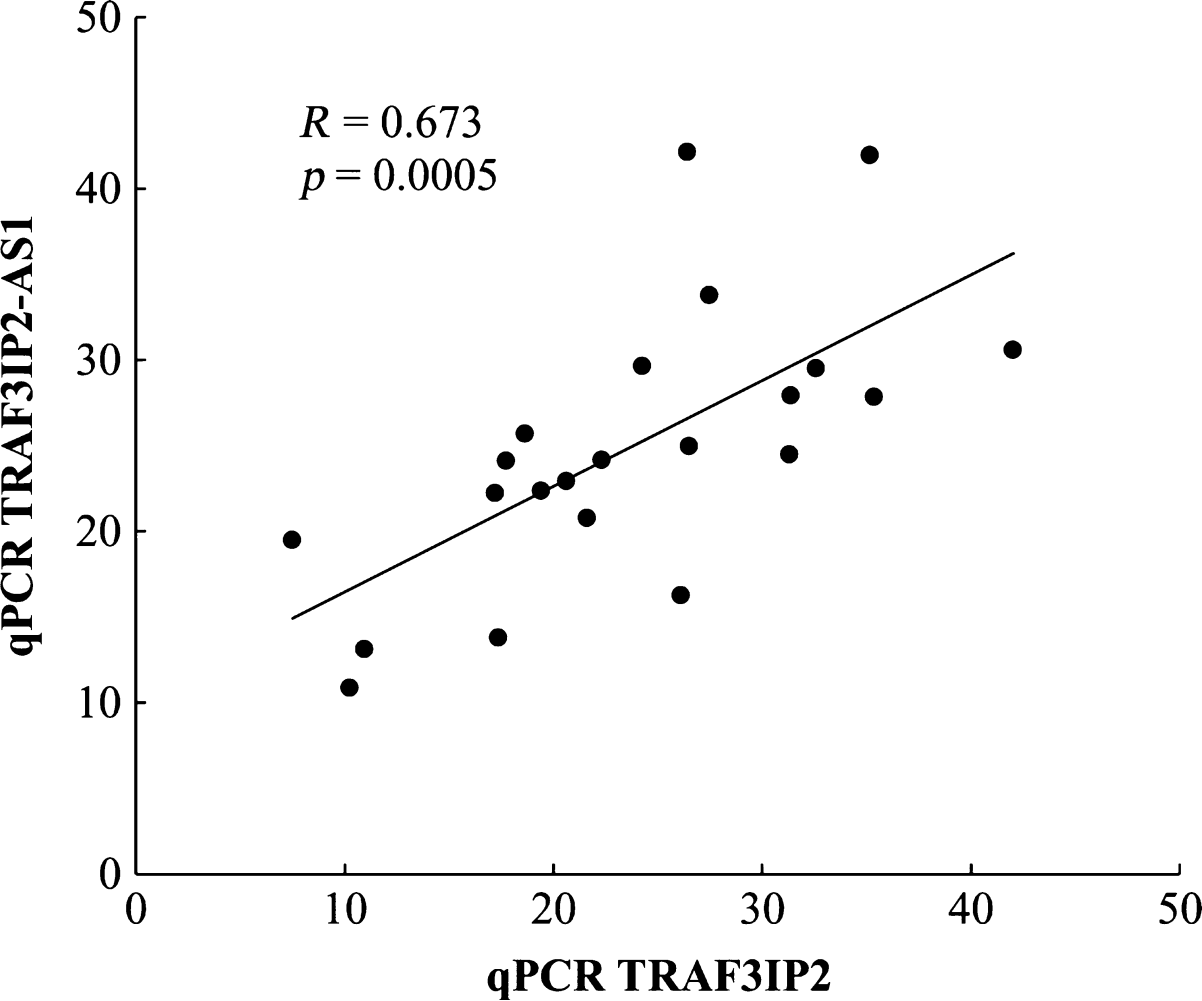
The expression of a protein‐coding transcript and lncRNA transcript from the TRAF3IP2 locus are significantly correlated. Pearson's correlation between TRAF3IP2 protein‐coding transcript and TRAF3IP2‐AS1 transcript abundances (as determined by qPCR) is shown. Primer sequences can be found in Table S1.

In contrast to LINC00162, TRAF3IP2‐AS1 transcript showed a surprisingly strong nuclear localization in DA cells (Fig. 2g and h). Int erestingly, the subcellular distribution of TRAF3IP2 protein‐coding transcript (from the strand opposite TRAF3IP2‐AS1) was quite distinct from that of TRAF3IP2‐AS1 transcript, being found throughout the nucleus, cytoplasm, and processes of DA neurons (Fig. 2i and j). As shown by qPCR, TRAF3IP2‐AS1 transcript abundance correlated significantly with the levels of TRAF3IP2 protein‐coding transcript (Fig. 3), consistent with a potential effect of TRAF3IP2‐AS1 on the expression of its cognate protein‐coding gene.

Given the dearth of functional data for most lncRNAs, potential functionality has been inferred through computational investigations of the co‐expression patterns of lncRNAs and protein‐coding genes across different conditions or tissues (Jiang *et al*. 2015). Our custom lncRNA microarray (Lipovich *et al*. 2012) included (as positive controls) probes for a number of protein‐coding neuroplasticity, stress‐response, and immediate early genes known to be up‐regulated in cocaine abusers' midbrains (Bannon *et al*. 2014). As shown in Table 3, the expression of these known cocaine‐responsive genes correlated significantly with expression of a subset of the differentially expressed lncRNAs, including RP11‐309G3.3, the lncRNA most up‐regulated in our dataset. It was perhaps noteworthy that the RP11‐309G3.3 gene lies immediately adjacent to an immediate early gene (*IER5*). In addition, our microarray also included probes for several DA cell phenotypic genes that are down‐regulated in cocaine abusers' midbrains (Bannon *et al*. 2014). In this study, the abundance of HOX antisense intergenic RNA myeloid 1, an lncRNA implicated in neurogenesis and brain development (Lin *et al*. 2011), was negatively correlated with the expression of transcripts encoding the DA transporter (−0.443; *p* < 0.05), the DA biosynthetic enzyme tyrosine hydroxylase (−0.503; *p* < 0.05), and the DA‐specifying transcription factor nurr1 (−0.469; *p* < 0.05). These correlative data suggest potential functional relationships between the expression of numerous cocaine‐responsive protein‐coding genes and some specific lncRNAs identified in this study. Furthermore, a global examination of all 32 lncRNAs (Table 2) for patterns of co‐expression with known protein‐coding genes across 19 human tissues (using the lncRNA2Function database; see Materials and methods), revealed a highly significant association with gene ontology terms related to synapse and neuron (cellular component), and transporter and channel activities (molecular function) (Table S3), further implicating these lncRNAs in the regulation of neural function.

**Table 3:** Correlations between differentially expressed lncRNAs and protein‐coding stress, neuroplasticity, and immediate early gene transcripts

	RP11‐309G3.3	RP11‐109G23.3	RPPH1	AC083843.1	RP11‐552F3.9	LINC00540
FOS	0.699***	0.606**	0.797***	0.775***	0.430*	−0.454*
FOSB	0.714***	0.622**	0.802***	0.778***	0.448*	−0.465*
JUN	0.804***	0.730***	0.848***	0.760***	0.483*	−0.485*
CEBPD	0.720***	0.717***	0.758***	0.559**	0.512*	−0.438*
ATF3	0.969***	0.917***	0.919***	0.611**	0.416	−0.531*
HSPA1A	0.959***	0.947***	0.886***	0.602**	0.358	−0.483*
BAG3	0.955***	0.966***	0.864***	0.519*	0.377	−0.413
GADD45B	0.710***	0.634**	0.828***	0.793***	0.541**	−0.456*
CDKN1A	0.848***	0.848***	0.775***	0.346	0.478*	−0.392
GADPH	−0.298	−0.379	−0.312	−0.363	−0.264	−0.019

Significantly correlated ***p < 0.001, **p < 0.01, *p < 0.05 (2‐tailed). GAPDH is a housekeeping gene included as a negative control.

## Discussion

The major goal of this study was to identify lncRNAs that are significantly dysregulated in the ventral midbrain of human cocaine abusers. Since they do not encode protein products, lncRNA transcripts constitute the final mediators of lncRNA gene function. Using an experimental design that incorporated parallel processing and quadruplicate hybridization of specimens from well‐matched subject pairs of cocaine fatalities and drug‐free control subjects (Table 1), followed by the application of various statistical, magnitude difference, and expression data filters (Lipovich *et al*. 2012), we identified 32 well‐annotated lncRNAs with clear differential expression in the midbrains of human cocaine abusers (Table 2). The robustness of the dataset obtained was confirmed by the successful validation (by qPCR) of differential lncRNA expression in every instance examined (Fig. 1).

A number of limitations associated with this study warrant mention. The application of stringent subject inclusion and exclusion criteria, and the careful matching of the cocaine‐abusing and control cohorts in terms of numerous demographic and sample quality parameters, limited the number of subjects available for study. The list of differentially expressed lncRNAs we identified by microarray is, in all likelihood, far from exhaustive; future RNA‐seq experiments involving larger cohorts and encompassing ongoing advances in lncRNA annotation, will undoubtedly extend the findings of this preliminary analysis. In addition, as the current experiments involved only cocaine abusers, other studies are needed to determine the extent to which these differentially expressed lncRNAs reflect changes common to all drug abusers versus cocaine‐specific effects. Previous studies of nucleus accumbens have identified both commonalities and differences in profiles of gene expression between cocaine and heroin abusers (Albertson *et al*. 2004, 2006; Michelhaugh *et al*. 2011). Furthermore, genomic studies in human and/or animal models are required to address the possibility that some differentially expressed lncRNAs might be associated with a predisposition to, rather than a response to, drug abuse. Finally, although the two lncRNAs selected for ISHH were subsequently shown to be expressed nearly exclusively within DA neurons (Fig. 2), the cellular locus of expression of the remaining lncRNAs was not examined; it is quite plausible that glia or non‐DA neurons contribute to the pattern of differential lncRNA expression observed in our microarray and qPCR experiments. Additional studies are clearly needed to advance our understanding of these issues.

As is the case for nearly all lncRNAs (Jiang *et al*. 2015), the biological functions of the cocaine‐responsive lncRNAs identified in this study are not currently understood. Computational investigations were therefore used to provide some preliminary insights into their potential functionality. As discussed, the lncRNA dataset as a whole (Table 2), based on the pattern of co‐expression with protein‐coding genes across human tissues, was very strongly associated with gene ontology terms related to neuronal function (Table S3). Furthermore, inclusion in our custom lncRNA microarray of probes for numerous protein‐coding genes that are up‐regulated (i.e., neuroplasticity, stress‐response, and immediate early genes) or down‐regulated (i.e., DA cell phenotypic genes) in cocaine abusers' midbrains (Bannon *et al*. 2014, 2015) allowed us to identify a specific subset of lncRNAs (Table 3) whose expression was significantly correlated with these known cocaine‐responsive genes. The potential functional relationship between these cocaine‐responsive lncRNAs and protein‐coding genes warrants further investigation.

Another interesting finding was the up‐regulation in cocaine abusers of the lncRNA TRAF3IP2‐AS1 (Table 2), and its positive correlation with the opposite strand protein‐coding transcript TRAF3IP2 (Fig. 3), despite their distinct subcellular localizations (Fig. 2). The exclusively nuclear localization of TRAF3IP2‐AS1 transcript and its lack of complementarity with TRAF3IP2 protein‐coding transcript sequence suggest a possible epigenetic effect of TRAF3IP2‐AS1 transcript on *TRAF3IP2* gene expression through alterations of chromatin state at this locus, as has been shown for some other antisense lncRNAs (Khorkova *et al*. 2014). Another lncRNA gene we found dysregulated in cocaine abusers, *PRKCQ‐AS1* (protein kinase C theta‐antisense 1) (Table 2) is antisense to the protein‐coding *PRKCQ* (protein kinase C theta) gene with which it shares a common promoter region. It is noteworthy that both TRAF3IP2 and PRKCQ proteins interact with other signaling molecules to activate the transcription factor nuclear factor kappa‐light‐chain‐enhancer of activated B cells (NF‐kB) (Chuang *et al*. 2011; Valente *et al*. 2013), as we have previously identified dysregulation of several NF‐kB‐associated genes in cocaine abusers' midbrains (Bannon *et al*. 2014) and NF‐kB signaling has been shown to regulate cocaine reward (Russo *et al*. 2009). TRAF3IP2‐AS1 and PRKCQ‐AS1 lncRNAs represent potential mediators of a disruption of NF‐kB signaling seen in cocaine abuse.

In summary, the current experiments represent, to our knowledge, the first profile of lncRNA dysregulation associated with human drug abuse. A small dataset of well‐annotated lncRNAs exhibiting robust differential expression in cocaine abusers' midbrains was identified. Examples of lncRNAs with DA cell‐specific expression, differential subcellular distribution, or covariance with known cocaine‐responsive protein‐coding genes were identified. In keeping with the emerging myriad roles of lncRNAs in brain development and some other CNS disorders (Michelhaugh *et al*. 2011; Modarresi *et al*. 2012; Pastori and Wahlestedt 2012; Ng *et al*. 2013; Clark and Blackshaw 2014; Punzi *et al*. 2014), we hypothesize that a number of the lncRNAs identified in this study mediate broader downstream changes in gene expression arising within the DA neurons of chronic drug abusers. Delineating the contributions of specific lncRNAs to the molecular processes underlying drug addiction will require experimental interventions in animal models, but could ultimately lead to the development of novel therapeutic approaches for the treatment of addiction.

## Supporting information


**Figure S1.** Criteria used for selection of lncRNAs for further analyses.
**Table S1.** Sequences of primers used for qPCR validation and of riboprobes used for *in situ* hybridization histochemistry.
**Table S2.** Absence of correlation between cocaine metabolite benzoylecgonine and differentially expressed lncRNAs from Table 2.
**Table S3.** Gene ontology terms associated with the lncRNA dataset in Table 2.
