# Supplementary material for: Identification of long noncoding RNAs dysregulated in the midbrain of human cocaine abusers
Source: J Neurochem. 2015 Sep 1;135(1):50–9. doi: 10.1111/jnc.13255 (PMC4830632; doi:10.1111/jnc.13255)
Supplement: Supplementary file 1 — Figure S1. Criteria used for selection of lncRNAs for further analyses. Table S1. Sequences of primers used for qPCR validation and of riboprobes used for in situ hybridization histochemistry. Table S2. Absence of correlation between cocaine metabolite benzoylecgonine and differentially expressed lncRNAs from Table 2. Table S3. Gene ontology terms associated with the lncRNA dataset in Table 2. [file JNC-135-50-s001.pdf]

Identification of long noncoding RNAs dysregulated in the midbrain of human cocaine abusers

Michael J. Bannon*, Candace L. Savonen*, Hui Jia[†], Fabien Dachet[‡], Steven D. Halter*, Carl J Schmidt[¶], Leonard Lipovich[†‡], and Gregory Kapatos*[†]

[*]Department of Pharmacology, Wayne State University School of Medicine

[†]Center for Molecular Medicine and Genetics, Wayne State University School of Medicine

[‡]Department of Neurology, Wayne State University School of Medicine, Detroit, Michigan, USA

[¶]Department of Pathology, University of Michigan, Ann Arbor, Michigan, USA.

Supplementary Information Figures and Tables

Figure S1. Criteria used for selection of lncRNAs for further analyses.

Table S1. Sequences of primers used for qPCR validation and of riboprobes used for in situ hybridization histochemistry.

Table S2. Absence of correlation between cocaine metabolite benzoylecgonine and differentially expressed lncRNAs from Table 2.

Table S3. Gene ontology terms associated with the lncRNA dataset in Table 2.

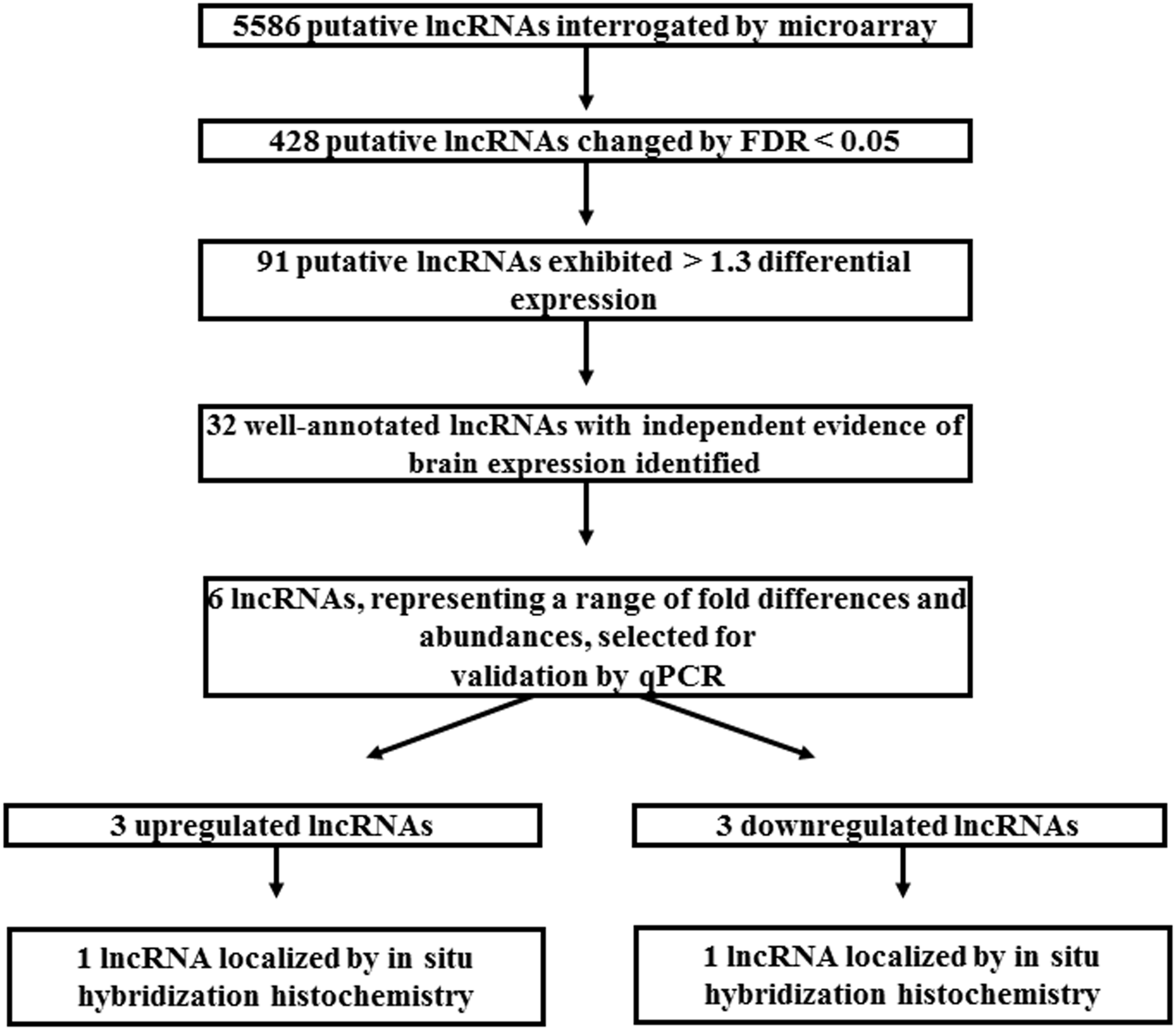

Supplementary Figure 1. Criteria used for selection for lncRNAs for further analyses.

Supplementary Table 1. Sequences of primers used for qPCR validation and of riboprobes used for in situ hybridization histochemistry.

RNA	Forward (5'-3')	Reverse (5'-3')	RefSeq ID
**LINC00162**	GTCCTTTTCCTTGAGTAGGG	AAACATTTCCTCTGTTTTCAC	NR_024089.2
**LINC01314**	GAAGGACAAACAGCCTCTGC	GGATTCTCAGGCAGTGCTTC	NR_120317.1
**LOC643764**	GCCTCTTTTTGTTGCTGTGA	GCAACCTGTGCCTTTGAAAT	NR_027378.1
**LOC100507534**	ACTAAGGAATCTGGGAGCTG	TGAATGGGGTAACCACTATG	NR_110649.1
**RP1-212P9.2**	ACAGCCTGGAAAGGAAGAAG	ACCACAGCAATGAAGTGGAC	BC042916.1*
**TRAF3IP2-AS1**	GCTGGGTACAGAGGTGATTC	AAATTAACCAGGTGTGGTGG	NR_34108.1<br>NR_34109.1
**TRAF3IP2**	GGGGATGTAGGCTAGAATTG	AGAGATGCTTTTGTCCATGT	NM_000164281.2<br>NM_001164283.2<br>NM_147686.3

	Riboprobe Sequences (5'-3')	RefSeq ID
**LINC00162**	AAACAUUUCCUCUGUUUUCACUCAUGACUGAGCUUUCCAUCGGCC<br>GUGGCCCCCACGUGUAGCAGGCACCUGCAGGCUGCCCAUCUUCAC<br>CACAUGUGCUCCCCACCUAAGCAAUGCAGAGAGGCCGCUGUGGGG<br>GUCGUCCCUACUCAAGGAAAAGGAC	NR_024089.2
**SLC6A3 (DAT)**	CAAGUGUCCUGCUUGGUUUAGCUGUGCAGAAGGUGAAAUGGAGG<br>AAACCACAAAUUCAUGCAAAGUCCUUUCCCGAUGCGUGGCUCCCA<br>GCAGAGGCCGUAAAUUGAGCGUUCAGUUGACACAUUGCACACACA<br>GUCUGUUCAGAGGCAU	NM_001044.4
**TRAF3IP2-AS1**	AAAUUAACCAGGUGUGGUGGUGCAUGCCUGUGGUCCCAACAAUA<br>UGGGAGGCUAAGGUGGGAGGAAGACCUGAUCCCAGGAGGGCAAG<br>GUAGUUGUAUGUCCCUGGGAAUCACCUCUGUACCCAGCUCAUGCC<br>CACCCAUGCUUACCCCAGGCACAUGUUCUCACCCU	NR_34108.1,<br>NR_34109.1
**TRAF3IP2**	AGAGAUGCUUUUGUCCAUGUCAUUUUAUAAAUGAGUCCCUGGAA<br>UCUAAGAAAAGUUAAAGGACUUGCUAAAGGUCAUACAGCUAAUU<br>GGUGGUAGAACCAAAAUUAGAACACAGACUACAAUUCUAGCCUAC<br>AUCCCC	NM_000164281.2<br>NM_001164283.2<br>NM_147686.3

*No associated NCBI RefSeq Gene ID.

Supplementary Table 2. Absence of correlation between cocaine metabolite benzoylecgonine and the differentially expressed lncRNAs from Table 2.

	**AC083843.1**	**AFAP1AS1**	**AP001505.9**	**HOTAIRM1**	**LINC00162**	**LINC00403**	**LINC00540**	**LINC00645**
Correlation Coefficient	-.144	.220	.270	-.402	.349	-.119	-.427	.229
p-value 2 tailed	.672	.517	.422	.221	.293	.728	.190	.499

	**RNF219-AS1**	**RP11-109G23.3**	**RP11-23P13.6**	**RP11-309G3.3**	**RP11-388C12.1**	**RP11-421016.2**	**RP11-49I11.1**	**RP11-539L10.3**
Correlation Coefficient	.085	.150	-.446	.248	-.346	-.533	-.397	-.342
p-value 2 tailed	.805	.660	.169	.462	.297	.092	.227	.303

	**LINC01010**	**LINC01314**	**LOC100507140**	**LOC100507534**	**LOC101929176**	**LOC400548**	**LOC643763**	**PRKCQ-AS1**
Correlation Coefficient	.698*	.180	.472	-.248	-.409	-.191	.160	.221
p-value 2 tailed	.017	.596	.142	.463	.211	.573	.638	.513

	**RP11-552F3.9**	**RP11-553L6.5**	**RP1-212P9.2**	**RP4-809F18.2**	**RPPH1**	**STX18-AS1**	**TRAF3IP2-AS1**	**WDR11-AS1**
Correlation Coefficient	-.297	.210	-.346	-.425	.094	.459	-.044	-.578
p-value 2 tailed	.375	.535	.298	.192	.783	.156	.899	.063

* $p < 0.05$ level (2-tailed).

Supplementary Table 3. Gene ontology terms associated with the lncRNA dataset in Table 2.

GO term name	P-value	Corrected P-value
**Cellular Component**		
synapse	5.5196E-19	3.2587E-15
synapse part	4.3166E-18	2.3361E-14
neuron part	1.4826E-15	6.8774E-12
cell projection	3.4645E-14	1.2500E-10
neuron projection	4.9304E-14	1.5290E-10
synaptic membrane	2.5252E-13	6.5598E-10
integral to plasma membrane	5.8535E-12	1.4621E-08
intrinsic to plasma membrane	6.8151E-11	1.4277E-07
postsynaptic membrane	1.0176E-10	2.0651E-07
ion channel complex	1.6452E-09	2.7397E-06
plasma membrane	2.2484E-09	3.6504E-06
cell projection part	2.8257E-09	4.4759E-06
cell periphery	2.9574E-09	4.5730E-06
synaptic vesicle	5.5280E-09	8.1592E-06
nucleosome	3.3163E-08	3.7133E-05
synaptic vesicle membrane	5.2706E-08	5.7048E-05
axon	8.0479E-08	8.1665E-05
acrosomal vesicle	1.3674E-07	1.3455E-04
plasma membrane part	2.9958E-07	2.7794E-04
chromatoid body	3.1370E-07	2.8694E-04
**Molecular Function**		
passive transmembrane transporter activity	7.9432E-15	3.2241E-11
channel activity	7.9432E-15	3.2241E-11
substrate-specific channel activity	1.7975E-14	6.8669E-11
ion channel activity	3.9326E-14	1.3442E-10
gated channel activity	2.4321E-13	6.5598E-10
ion gated channel activity	2.4321E-13	6.5598E-10
metal ion transmembrane transporter activity	2.2322E-10	4.3929E-07
GABA receptor activity	3.2193E-10	6.1491E-07
voltage-gated ion channel activity	7.3777E-10	1.3309E-06
voltage-gated channel activity	7.3777E-10	1.3309E-06
GABA-A receptor activity	1.4608E-09	2.4965E-06
ion transmembrane transporter activity	5.4990E-09	8.1592E-06
voltage-gated cation channel activity	1.0745E-08	1.4241E-05
inorganic cation transmembrane transporter activity	2.5887E-08	3.0021E-05
cation channel activity	2.5625E-08	3.0021E-05
transmembrane transporter activity	2.8241E-08	3.2177E-05
monovalent inorganic cation transmembrane transporter	7.7443E-08	8.1037E-05
sodium ion transmembrane transporter activity	1.2624E-07	1.2613E-04
substrate-specific transmembrane transporter activity	2.0601E-07	1.9675E-04
transmembrane signaling receptor activity	6.4895E-07	5.3348E-04

Results obtained using the LncRNA2Function database (See Materials and Methods). Top 20 terms are shown.
